# Adefovir anticancer potential: Network pharmacology, anti-proliferative and apoptotic effects in HeLa cells

**DOI:** 10.17305/bb.2025.12058

**Published:** 2025-03-18

**Authors:** Muzammal Mateen Azhar, Tahir Maqbool, Fatima Ali, Awais Altaf, Muhammad Atif, Zulfiqar Ali, Zahid Habib Qureshi, Muhammad Naveed, Tariq Aziz, Rania Ali El Hadi Mohamed, Fakhria A Al-Joufi, Maher S Alwethaynani

**Affiliations:** 1 Centre for Research in Molecular Medicine, Institute of Molecular Biology and Biotechnology, The University of Lahore, Lahore, Pakistan; 2 Department of Pharmacy, The University of Lahore, Lahore, Pakistan; 3 Lahore Medical and Dental College, Lahore, Pakistan; 4 Multan Medical and Dental College, Multan, Pakistan; 5 Department of Biotechnology, Faculty of Science and Technology, University of Central Punjab, Pakistan; 6 Laboratory of Animal Health Food Hygiene and Quality University of Ioannina, Arta, Greece; 7 Department of Biology, College of Science, Princess Nourah bint Abdulrahman University, P. O. Box 84428, Riyadh, 11671, Saudi Arabia; 8 Department of Pharmacology, College of Pharmacy, Jouf University, 72341 Aljouf, Saudi Arabia; 9 Department of Clinical Laboratory Sciences, College of Applied Medical Sciences, Shaqra University, Alquwayiyah, Riyadh, Saudi Arabia

**Keywords:** Adefovir, HeLa cells, apoptosis, angiogenesis, network pharmacology

## Abstract

Cervical cancer presents a significant healthcare challenge due to recurrent disease and drug resistance, highlighting the urgent need for novel therapeutic strategies. Network pharmacology facilitates drug repurposing by elucidating multi-target mechanisms of action. Adefovir, an acyclic nucleotide analog, has shown promising potential in cervical cancer treatment, particularly in HeLa cells. *In vitro* studies have demonstrated that adefovir inhibits HeLa cell proliferation by enhancing apoptosis while maintaining a low cytotoxicity profile at therapeutic concentrations, making it an attractive candidate for further exploration. A combined network pharmacology and *in vitro* study was conducted to investigate the molecular mechanism of adefovir against cervical cancer. Potential gene targets for adefovir and cervical cancer were predicted using database analysis. Hub targets were identified, and protein-protein interaction (PPI) networks were constructed. Molecular docking assessed adefovir’s binding affinity to key targets. *In vitro* cytotoxic assays, including 3-(4,5-Dimethylthiazol-2-yl)-2,5-diphenyltetrazolium bromide (MTT) and crystal violet assays, were performed using 96-well plates to evaluate anti-proliferative effects in HeLa cells. Apoptosis was assessed via p53 immunocytochemistry enzyme-linked immunosorbent assay (ELISA), while vascular endothelial growth factor ELISA (VEGF ELISA) was used to measure cell proliferation. Venn analysis identified 144 common targets between adefovir and cervical cancer. Network analysis revealed key hub targets involved in oncogenic pathways. Molecular docking demonstrated strong binding between adefovir and mitogen-activated protein kinase 3 (MAPK3) and SRC proteins. *In vitro*, adefovir significantly suppressed HeLa cell viability, with an inhibitory concentration 50 (IC_50_) of 7.8 µM, outperforming 5-Fluorouracil (5-FU). Additionally, it induced apoptosis via p53 activation and inhibited cell proliferation through VEGF suppression. These integrated computational and experimental findings suggest that adefovir exerts multi-targeted effects against cervical cancer. Its promising preclinical efficacy warrants further investigation as a potential alternative therapy.

## Introduction

Cervical cancer’s high morbidity and mortality rates make it a persistent global public health concern [1]. Despite advances in surgical and chemotherapeutic interventions, treatment outcomes remain suboptimal, with high risks of recurrence, drug resistance, and adverse effects posing significant challenges [2]. This underscores the urgent need for novel therapeutic strategies with improved efficacy and safety profiles. Drug repositioning offers a promising approach by identifying new clinical indications for approved drugs, thereby shortening development timelines [3]. Network pharmacology has emerged as a powerful computational framework for gaining mechanistic insights into drug actions and facilitating drug repositioning opportunities [4]. It leverages omics datasets, pathway information, and systems modeling approaches to provide a holistic view of compound interactions at the molecular, pathway, and network levels [5]. These insights can reveal multi-targeted mechanisms and uncover unanticipated therapeutic applications. Several network pharmacology studies have successfully identified repurposing candidates for various cancers [6]. The FDA has approved adefovir dipivoxil, an orally bioavailable prodrug of adefovir, for the treatment of chronic hepatitis B [7]. Beyond its antiviral activity, recent studies have reported adefovir’s anti-proliferative effects in hepatocellular carcinoma and prostate cancer [8]. However, its potential against cervical cancer remains unexplored. Given the involvement of oncogenic pathways such as MAPK/PI3K signaling in multiple cancers [9], adefovir represents an attractive candidate for network-based evaluation against cervical cancer.

This study aims to investigate the molecular mechanisms and therapeutic potential of adefovir against cervical cancer using a comprehensive network pharmacology experimental workflow. Integrated computational target prediction, pathway analysis, molecular docking, and experimental validation will be employed to provide an in-depth understanding of adefovir’s anticancer properties.

## Materials and methods

### Network pharmacology analysis

Molecular docking was performed using Molecular Operating Environment (MOE) 2019 (Chemical Computing Group, Montreal, Canada). Molecular visualization was conducted with Discovery Studio Client 2021 (BIOVIA, Dassault Systèmes, San Diego, CA, USA). Hub gene identification and compound-target network generation were carried out using Cytoscape v3.10.3 (Institute for Systems Biology, Seattle, WA, USA), while protein–protein interaction (PPI) network analysis was conducted via the STRING app (Swiss Institute of Bioinformatics, Lausanne, Switzerland). Functional enrichment analysis was performed using the DAVID Database (Laboratory of Human Retrovirology and Immunoinformatic, Frederick, MD, USA). Enrichment graphs were generated with the SR Plot Platform (Bioinformatics and Statistics Hub, online platform). Statistical analysis of *in vitro* experiments was conducted using GraphPad Prism (GraphPad Software, Boston, MA, USA).

### Adefovir-associated target prediction

Adefovir’s canonical SMILES were obtained from the PubChem database [10]. Potential targets were identified using the WAY2DRUG (DIGEP-Pred 2.0) [11] and STITCH [12] databases, based on structural similarity, pharmacophore mapping, and pathway analysis. Targets were prioritized using confidence scores (e.g., >0.7 in STITCH). To develop the adefovir-related target database, redundant entries were removed, and target names were converted to human gene names using the UniProt database [13].

### Cervical cancer-associated target prediction

Genes linked to cervical cancer were identified through a search of several open-access online databases. The screened databases included the Therapeutic Target Database, which catalogs molecular targets of drug therapies, as well as OMIM (Online Mendelian Inheritance in Man) [14] and GeneCards (Human Gene Database) [15].

### Venn analysis

A Venn diagram was generated using the bioinformatics software Venny 2.1.0 to visualize the overlap between gene sets identified in this study. Venny is a tool designed for comparing lists of genes or other items and generating Venn diagrams [16].

### Enrichment analysis of potential targets using KEGG Pathways and Gene Ontology (GO)

GO analysis is a widely used method for interpreting large genomic and transcriptomic datasets. In this study, GO and pathway enrichment analyses were performed on identified target genes to explore their functional roles [17]. The target genes were classified into three GO categories based on their biological roles: biological processes (BPs), cellular components (CCs), and molecular functions. A common online tool was used to analyze gene involvement in these categories, organizing them based on BPs, cellular localization, and functional annotation to better understand their roles. The study employed DAVID’s GO functional classification and Kyoto Encyclopedia of Genes and Genomes (KEGG) pathway mapping features. Enriched GO terms and KEGG pathways with a *P* value cutoff of 0.05 were selected for further analysis [18]. Significantly enriched GO terms and pathways (*P* < 0.05) were identified and visualized. This threshold ensured that only the most statistically relevant results were considered for further examination and representation. The SRplot platform was used to generate bubble charts, demonstrating its effectiveness as an online data analysis tool [19].

### PPIs and network analysis

PPIs refer to the ability of proteins to form complexes through non-covalent bonds between two or more protein molecules [20]. The common targets identified using a Venn diagram were analyzed in the STRING database to assess the relationship between adefovir and cervical cancer targets [21]. To better understand how the potential target genes interact at the molecular level, a PPI network analysis was conducted, focusing specifically on human protein interactions within Homo sapiens. Cytoscape was used to generate and visualize the PPI network, with the CytoHubba plugin employed to identify key genes based on their number of interactions (degree centrality). Higher-degree nodes, which play critical roles in network connectivity, were highlighted. Additionally, Cytoscape was used to construct networks linking identified drugs and targets to examine potential therapeutic mechanisms related to cervical cancer. Nodes were colored and shaped to differentiate between drugs and target genes, while edges represented PPIs [22].

### Molecular docking

#### Structures of target proteins

The 3D structures of SRC (PDB ID: 1Y57, 1.91 Å resolution), ABL1 (PDB ID: 1EIP, 2.10 Å resolution), PIK3CA (PDB ID: 4OVU, 2.96 Å resolution), PIK3R1 (PDB ID: 1H9O, 1.79 Å resolution), and mitogen-activated protein kinase 3 (MAPK3) (PDB ID: 4H3Q, 2.2 Å resolution) were obtained in Protein Data Bank (PDB) format from the Research Collaboratory for Structural Bioinformatics (RCSB) [23]. The MOE Protonate-3D module was used to preprocess these protein structures for docking analysis. This preprocessing included the removal of co-crystallized ligands, water molecules, and other heteroatoms, as well as the addition of missing hydrogen atoms and optimization of side-chain orientations. To minimize energy and resolve any steric conflicts or structural imperfections, the protein structures were further refined using the AMBER99 force field [24].

### Ligand preparation

The ligand structure was obtained from the PubChem database hosted by NCBI. Using Biovia Discovery Studio, the structure was converted into PDB format for further analysis. This conversion enabled the visualization and exploration of the ligand’s 3D structure from public databases [25]. MOE then preprocessed the ligand molecule by removing counterions and salts to generate the appropriate protonation state. Finally, the ligand’s energy was minimized using the MMFF94x force field to obtain its most stable conformation.

### Active binding site prediction of target proteins

The potential binding sites of the target proteins were computationally predicted using CASTp software [26].

### Molecular docking

MOE version 2019.0102 was used for molecular docking and scoring. The docking study was performed on ligands against various protein targets and visualized using the BIOVIA Discovery Studio Visualizer. First, the ligand database was created in MOE and converted into a Microsoft Access database file (MDB format). The input files were then uploaded for docking analysis, utilizing MOE’s default docking algorithm with 100 ligand conformations. The docking process employed the Triangle Matcher algorithm, with London dG and GBVI/WSA dG as scoring functions. The best-docked result was selected based on conformational visualization in BIOVIA [27].

### *In vitro* anti-proliferative analysis

#### HeLa cell lines

The HeLa cervical cancer cell line was obtained from the Cell and Tissue Culture Laboratory at the University of Lahore and stored in cryovials in liquid nitrogen. The study was approved by the Institutional Research Ethics Committee of the Department of Pharmacology, Faculty of Pharmacy, University of Lahore, Pakistan.

### Treatment

Adefovir (CAS number 106941-25-7) was purchased from Merck. The powdered drug was dissolved in sterile PBS to prepare a 1 M stock solution (2.73 g in 10 mL of PBS). A cell viability assay was conducted to determine the optimal concentration of adefovir. Different dilutions (1 µM, 3 µM, 5 µM, and 10 µM) were prepared in complete DMEM media from the 1 M stock solution. HeLa cervical cancer cells were plated in a 96-well cell culture plate and incubated overnight at 37 ^∘^C to allow adherence. The growth media was then removed, and the cells were rinsed with 1X PBS before treatment. Various concentrations of adefovir were introduced into the wells [28].

### Study design

HeLa cells were divided into the following groups (*n* ═ 3 in each group):

1. Control: Complete DMEM medium.
2. 5-FU (50 µM): 50 µM of 5-Fluorouracil in complete DMEM medium.
3. Adefovir (1 µM): 1 µM of adefovir in complete DMEM medium.
4. Adefovir (3 µM): 3 µM of adefovir in complete DMEM medium.
5. Adefovir (5 µM): 5 µM of adefovir in complete DMEM medium.
6. Adefovir (10 µM): 10 µM of adefovir in complete DMEM medium.

### Cell viability assay

MTT and crystal violet (CV) assays were used to assess the viability of treated HeLa cells. The cells were cultured in 96-well plates, and their viability was evaluated across various doses of adefovir.

### MTT assay

The cells were rinsed with 1X PBS and incubated for 3–4 h in a mixture of DMEM growth media (100 µL) and MTT reagent (25 µL). During this time, metabolically active cells reduced MTT into insoluble purple formazan crystals. The crystals were then solubilized using a 10% sodium dodecyl sulfate solution. Absorbance was measured at 570 nm using a microplate reader to quantify formazan levels, which correlate with cell viability. Percent viability was calculated by comparing the average absorbance values of treated samples to those of untreated control wells [28].

### CV assay

Treated cells were rinsed with 1X PBS and stained with a 0.1% CV dye solution for 15 min at room temperature. The cells were then thoroughly rinsed with PBS, taking care to avoid dislodging them from the bottom of the wells. To solubilize the incorporated dye, 100 µL of 1% sodium dodecyl sulfate was added, followed by incubation for 5–10 min at room temperature. Absorbance at 595 nm was measured using a microplate reader to quantify the dissolved CV, which directly correlates with the number of adherent cells [28].

### Dead cells detection

A trypan blue assay was performed to detect dead cells. Trypan blue staining distinguishes live cells from dead ones. Briefly, cells treated with adefovir were rinsed with 1X PBS and then stained with trypan blue (Cat. No. T6146). Blue-stained cells were identified as dead and counted using a compound microscope [28].

**Figure 1.**
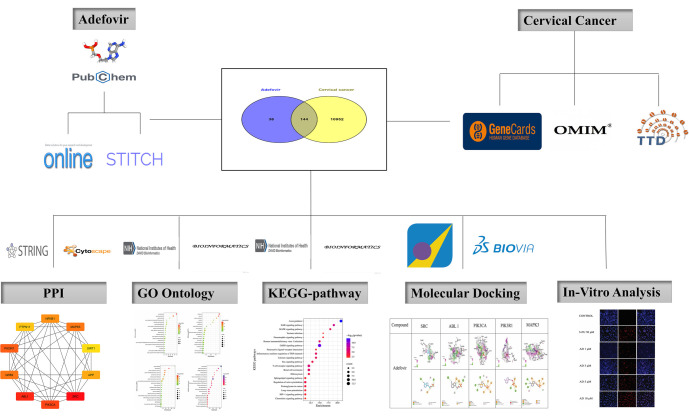
**Overview of the Adefovir and cervical cancer analysis workflow.** This figure illustrates the integrated methodology employed to examine the relationship between adefovir and cervical cancer. The central Venn diagram displays the overlap of genes associated with both adefovir and cervical cancer, sourced from databases such as PubChem, GeneCards, OMIM, and TTD. The workflow branches into various analytical methods, including protein-protein interaction (PPI) analysis utilizing tools like STRING and Cytoscape, Gene Ontology (GO) analysis, KEGG pathway analysis, molecular docking studies, and *in vitro* analysis. Each method is visually represented, showcasing a comprehensive strategy to elucidate the connections between adefovir and cervical cancer, ultimately providing insights into potential therapeutic implications. PPI: Protein-protein interaction; GO: Gene Ontology; KEGG: Kyoto Encyclopedia of Genes and Genomes.

### Enzyme-linked immunosorbent assay (ELISA) for *p53* and vascular endothelial growth factor (VEGF)

The Bioassay Technology Laboratory ELISA kit was used to evaluate inflammation and apoptosis. All reagents, standard solutions, and samples were prepared according to the manufacturer’s instructions. Before use, reagents were brought to room temperature, and the experiment was conducted under these conditions. The required number of strips for the assay was determined, and they were placed into the frame for use, while any unused strips were stored at 2—8 ^∘^C. A 50 µL standard solution was added to the standard wells, and 40 µL of the sample was added to the sample wells. This was followed by the addition of 10 µL of p53 (apoptosis) and VEGF (angiogenesis) antibodies to the sample wells. After incubating the sample and standard wells, 50 µL of streptavidin-HRP was added to both (excluding blank or control wells). The mixture was thoroughly mixed, sealed with parafilm, and incubated for the designated period. Following incubation, the well plate was washed with wash buffer. Then, 50 µL of substrate solution A and 50 µL of substrate solution B were sequentially added. The plate was incubated at 37 ^∘^C for 10 min. After incubation, 50 µL of stop solution was added, immediately turning the blue color to yellow. The optical density at 450 nm was measured within 10 min of adding the stop solution.

### Immunofluorescence with DAPI and PI

The cells were cultured on coverslips and fixed with 4% paraformaldehyde for 15 min at room temperature. After fixation, they were permeabilized with 0.1% Triton X-100 for 10 min. For nuclear staining, the cells were incubated with DAPI (1 µg/mL) to visualize nuclei, followed by PI (1 µg/mL) for 10 min to stain dead or membrane-compromised cells. After staining, excess dye was removed by washing with PBS. Fluorescence microscopy images were captured using filters for DAPI (blue) and PI (red), and the coverslips were mounted with a suitable mounting medium.

### Ethical statement

The ethical approval for this study was granted by the Institute of Molecular Biology and Biotechnology at The University of Lahore, Punjab, Pakistan.

### Statistical analysis

Data from three biological replicates are presented as mean ± SD and analyzed using one-way ANOVA followed by Tukey’s multiple comparison test. All statistical analyses were performed using GraphPad Prism 8.0, with a significance threshold of *P* < 0.05. Significance in the graph is indicated as follows: *** for *P* ≤ 0.001, ** for *P* ≤ 0.01, and * for *P* ≤ 0.05.

## Results

### Graphical representation of word

The graphical representation of research work (Figure 1).

### Identification of potential targets of Adefovir and cervical cancer-related genes

**Figure 2.**
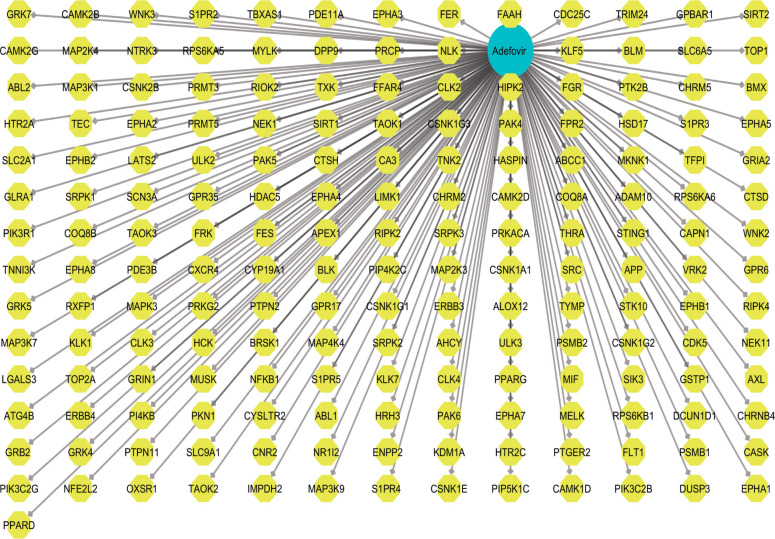
**Adefovir drug target network.** This network illustrates the interactions between adefovir and its potential drug targets. The central node represents adefovir, with surrounding nodes indicating various proteins or genes involved in its mechanism of action. Edges connecting the nodes denote the interactions between adefovir and these targets. Highlighted nodes signify key proteins that may play significant roles in the pharmacological effects of adefovir, particularly regarding anticancer activity.

**Figure 3.**
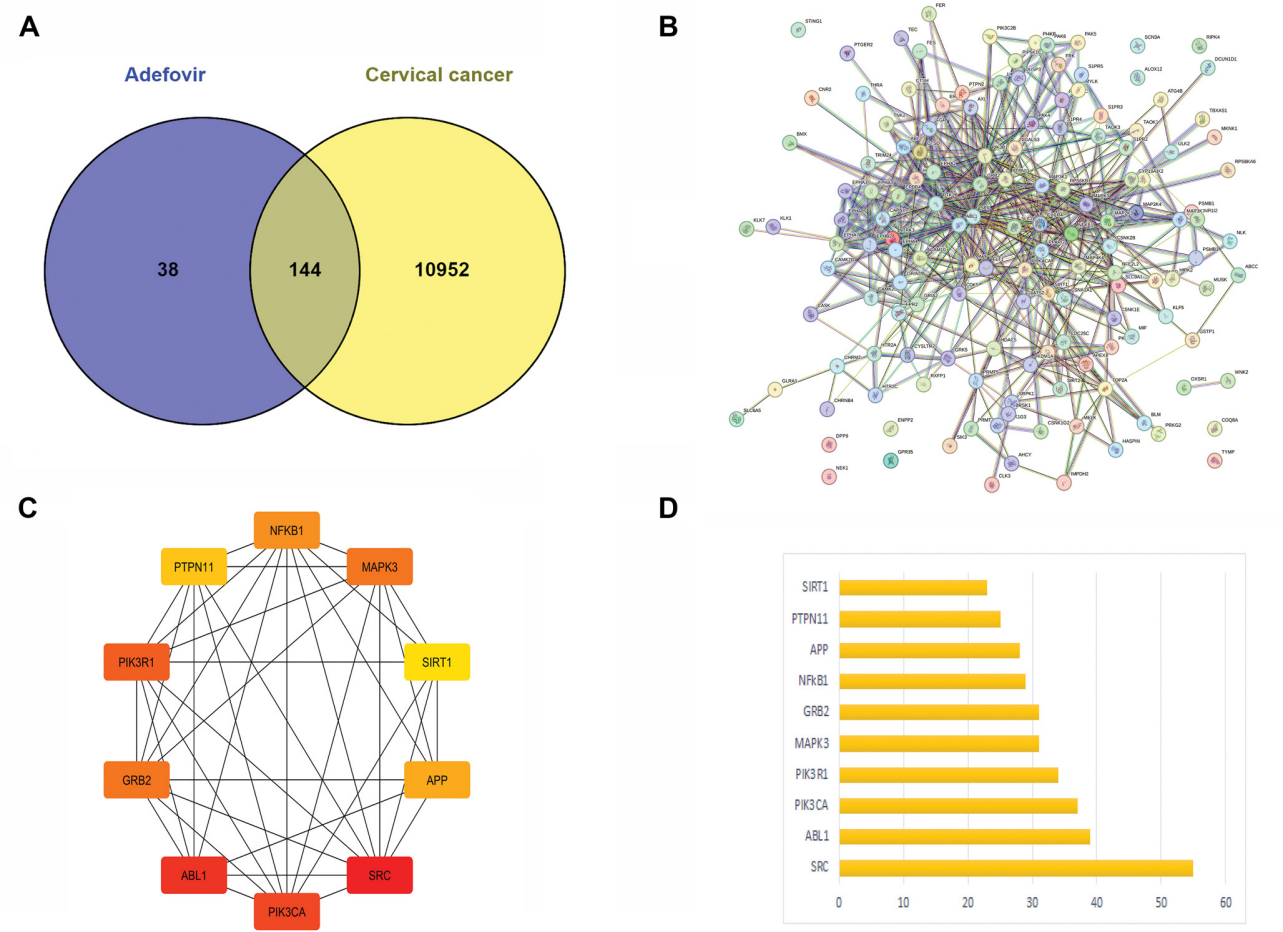
**Analysis of Adefovir’s role in cervical cancer.** (A) Venn diagram illustrating the overlap between adefovir-related genes and those associated with cervical cancer, identifying 144 common genes; (B) Interaction network of proteins associated with both adefovir and cervical cancer, where nodes represent proteins and edges denote interactions, highlighting the complex relationships among them; (C) Focused network showcasing key proteins (e.g., NFKB, SIRT1) that interact with both adefovir and cervical cancer pathways, emphasizing adefovir's potential role in modulating these interactions; (D) Bar graph depicting the frequency of interactions for selected proteins within the network, underscoring their importance in the biological context.

Adefovir’s potential targets were predicted using the WAY2DRUG and STITCH databases, identifying 182 candidate targets (Figure 2). Together with adefovir, these targets formed a compound-target network with 183 nodes, built using Cytoscape version 3.9.1, and 182 interactions represented as edges. Nodes for antiviral targets were colored blue, while those for other targets were colored yellow. The CytoHubba plugin of Cytoscape was used to analyze the network and determine the degree centrality (182), maximum neighborhood component (1), maximum clique centrality (182), closeness centrality (182), and betweenness centrality (32,942) for adefovir. In addition, 11,096 genes related to cervical cancer were identified from Genecards, OMIM, and the therapeutic target database. A Venn diagram was used to compare target genes associated with compounds and those linked to cervical cancer, resulting in the identification of 144 common targets (Figure 3A). Based on the assumption that these targets were significant, further analysis was conducted.

### Interaction of protein with other proteins

To explore PPIs among potential candidates of interest, 144 proteins were analyzed using the online database STRING version 11.5. This database generated an initial protein network linking the genes. The resulting file in TSV format was then downloaded for further analysis of the interaction data. The TSV file generated from STRING was imported into Cytoscape v3.9.1 to build and visualize the protein interaction network, as shown in Figure 3B. To identify central genes, the CytoHubba degree scoring tool in Cytoscape was used to rank the top 10 hub genes. The genes with the highest degree, including SRC (55), ABL1 (39), PIK3CA (37), PIK3R1 (34), MAPK3 (31), GRB2 (31), NFkB1 (29), APP (28), PTPN11 (25), and SIRT1 (23), were selected for further analysis (Figure 3C and 3D).

### Pathways and GO enrichment analysis

**Figure 4.**
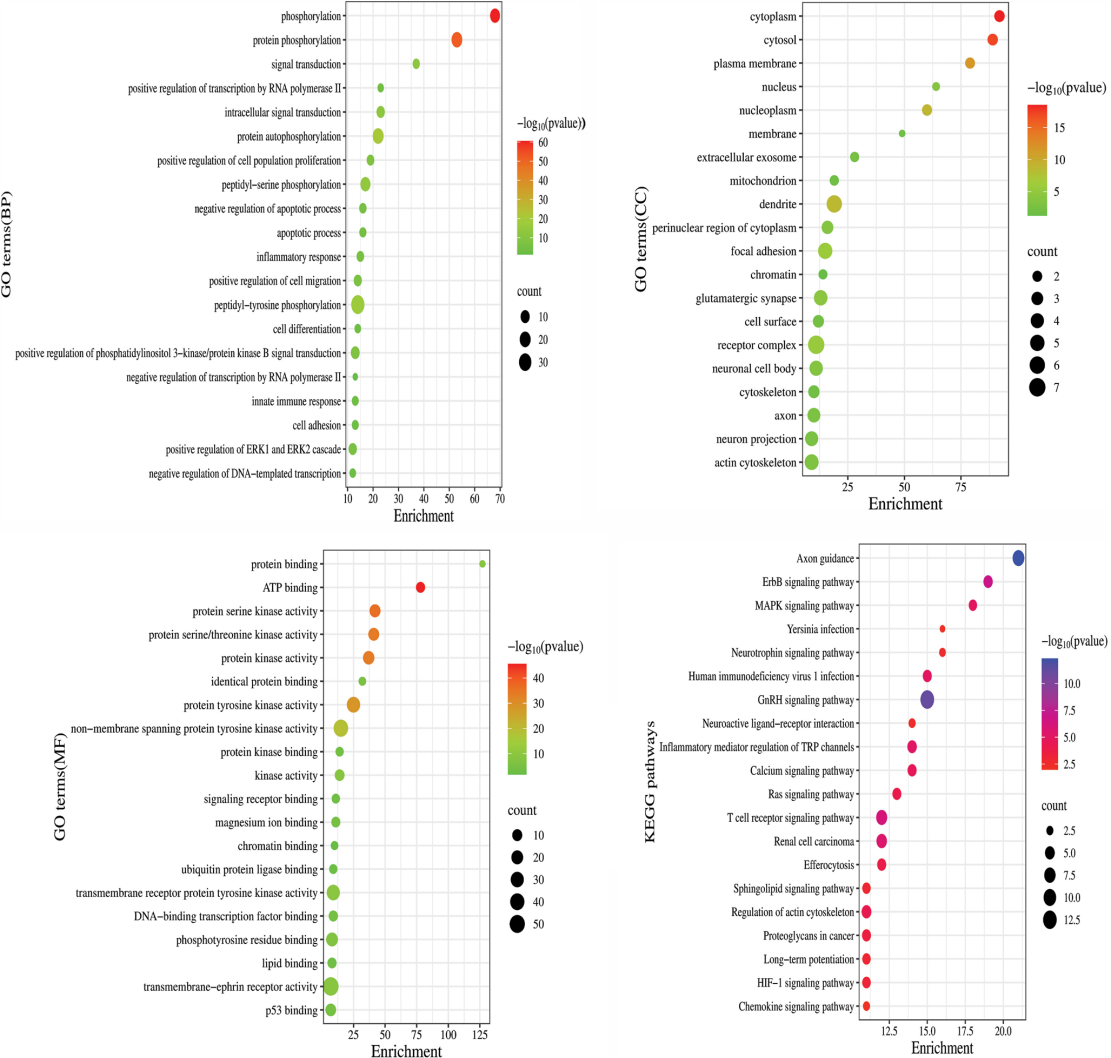
**Gene Ontology (GO) analysis of enrichment presents four panels illustrating the enrichment of different GO categories based on gene expression data.** The top left panel focuses on biological processes, highlighting significant enrichments such as “positive regulation of cellular process” and “negative regulation of metabolic process.” Each bubble represents a GO term; bubble size indicates the number of genes involved, while color reflects the significance level (−log10(*P* value)). The top right panel depicts cellular component GO terms, showcasing enrichments in categories like “membrane” and “cytoplasm,” maintaining the same bubble size and color coding. The bottom left panel is dedicated to molecular function GO terms, including “protein binding” and “ATP binding,” emphasizing the functional roles of the analyzed genes. Finally, the bottom right panel presents KEGG pathway analysis, illustrating significant pathways such as “MAPK signaling” and “DNA replication,” with bubble sizes based on gene count and colors indicating statistical significance. This comprehensive analysis underscores the biological relevance of the genes under investigation.

**Figure 5.**
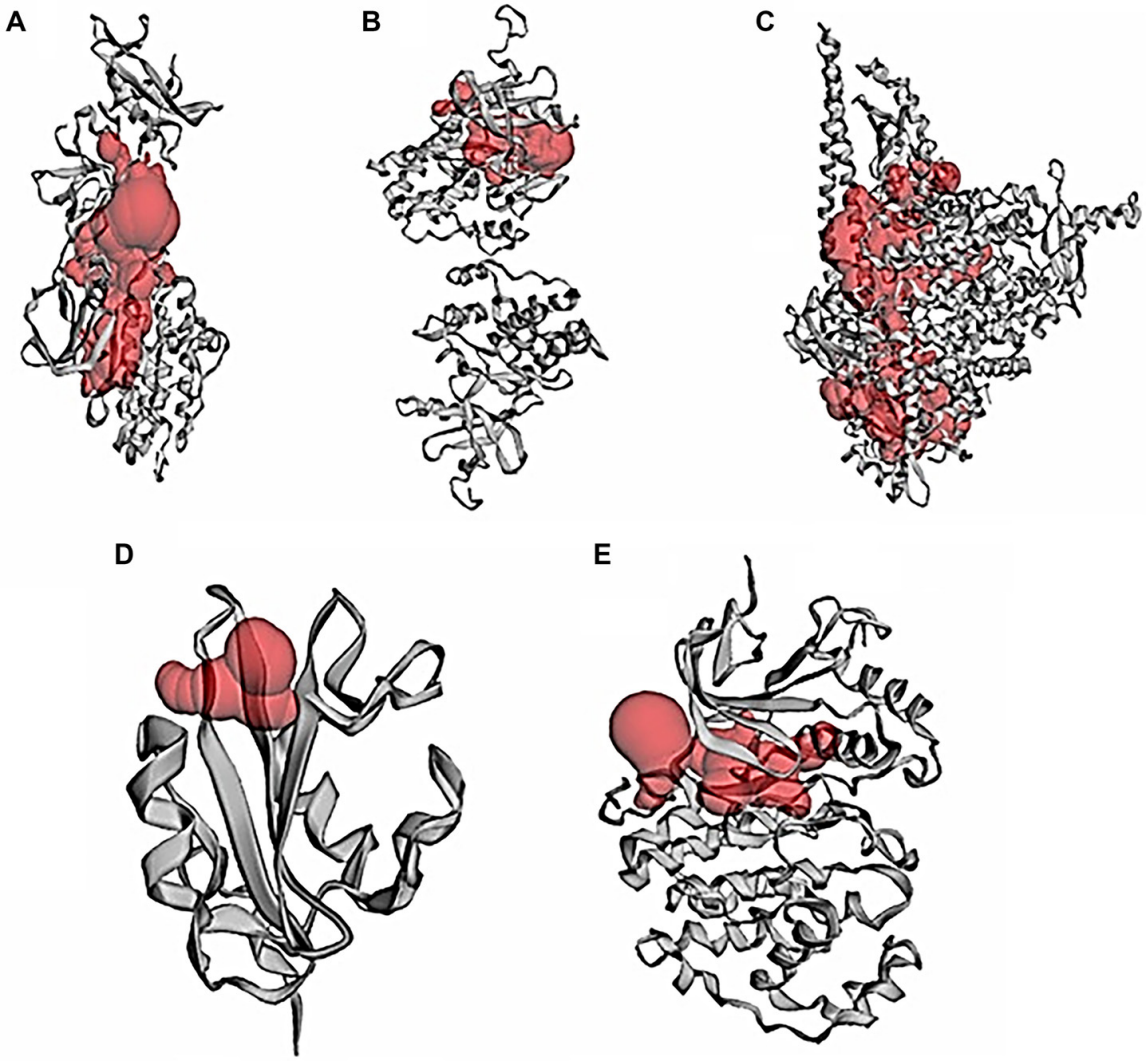
**The active site of target proteins was obtained from the CASTp server.** (A) SRC; (B) ABL1; (C) PIK3CA; (D) PIK3R1; and (E) MAPK3. MAPK3: Mitogen-activated protein kinase 3.

A total of 279 important BPs, 68 cellular constituents, 87 molecular activities, and 97 keywords related to KEGG pathways were available in the DAVID database. The target genes are primarily involved in processes, such as phosphorylation, protein phosphorylation, peptidyl-tyrosine phosphorylation, and others, as indicated by the BPs. The CCs are mainly associated with receptor complexes, dendrites, focal adhesions, and other structures (Figure 4). Molecular functions (Figure 5) highlight genes involved in processes, such as transmembrane–ephrin receptor activity, protein tyrosine kinase activity, non-membrane spanning tyrosine kinase activity, among others.

### Active binding sites of target proteins

**Figure 6.**
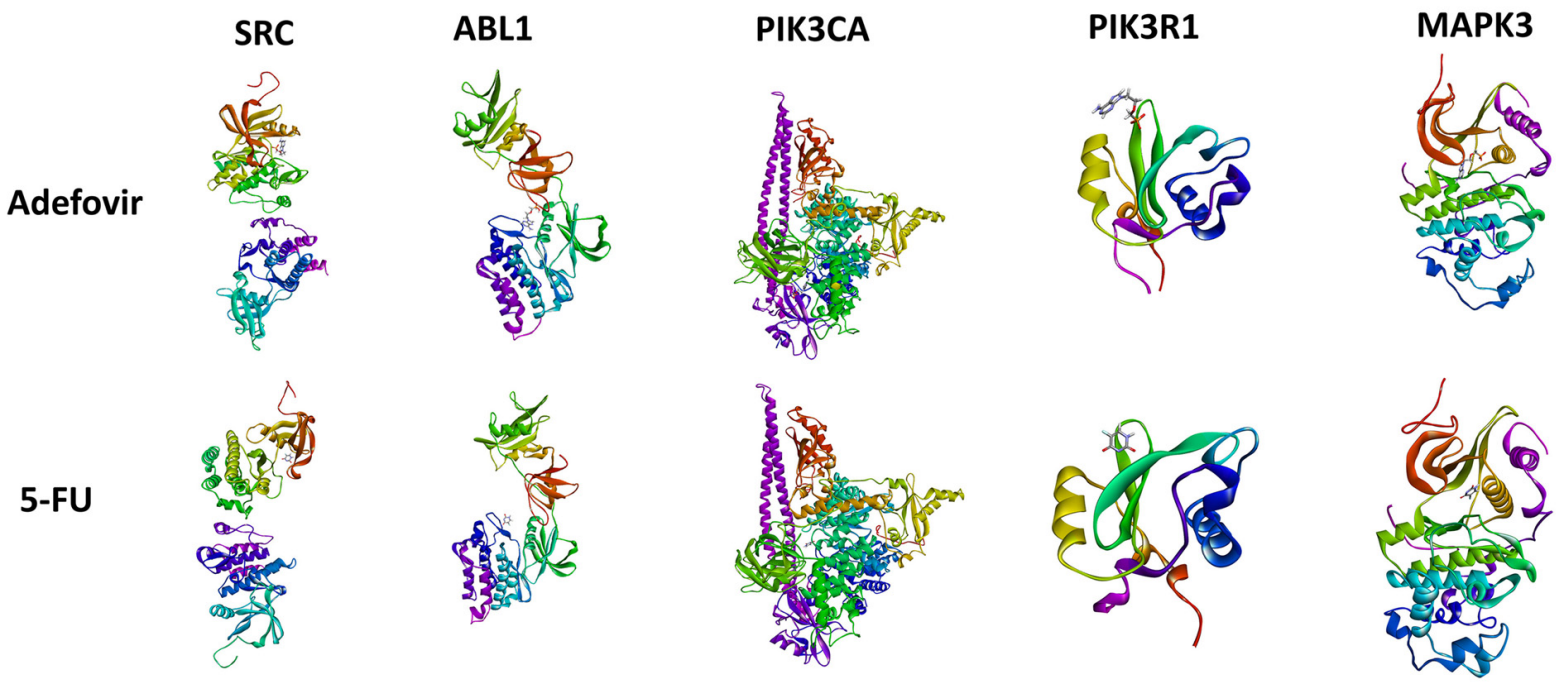
**3-dimensional configuration and compound interactions with different proteins.** MAPK3: Mitogen-activated protein kinase 3; 5-FU: 5-Fluorouracil.

The active binding sites of the target proteins were predicted using CASTp analysis. The findings are summarized in Table 1 and Figure 6, which detail the number of binding pockets, their surface areas, and the volumes of each pocket.

**Table 1:** Binding pockets, area, volume, color, and style of the top pocket of all proteins

**Sr no**	**Protein**	**Protein (PDB)**	**Binding pockets**	**Area (SA)**	**Volume (SA)**	**Negative volume color**	**Representation style**
1	SRC	1Y57	58	2217.453	3266.202	Red	Cartoon
2	ABL1	1IEP	64	1096.316	852.391	Red	Cartoon
3	PIK3CA	40VU	197	8023.264	7885.856	Red	Cartoon
4	PIK3R1	1H9O	14	67.140	29.788	Red	Cartoon
5	MAPK3	4H3Q	59	579.051	419.707	Red	Cartoon

**Figure 7.**
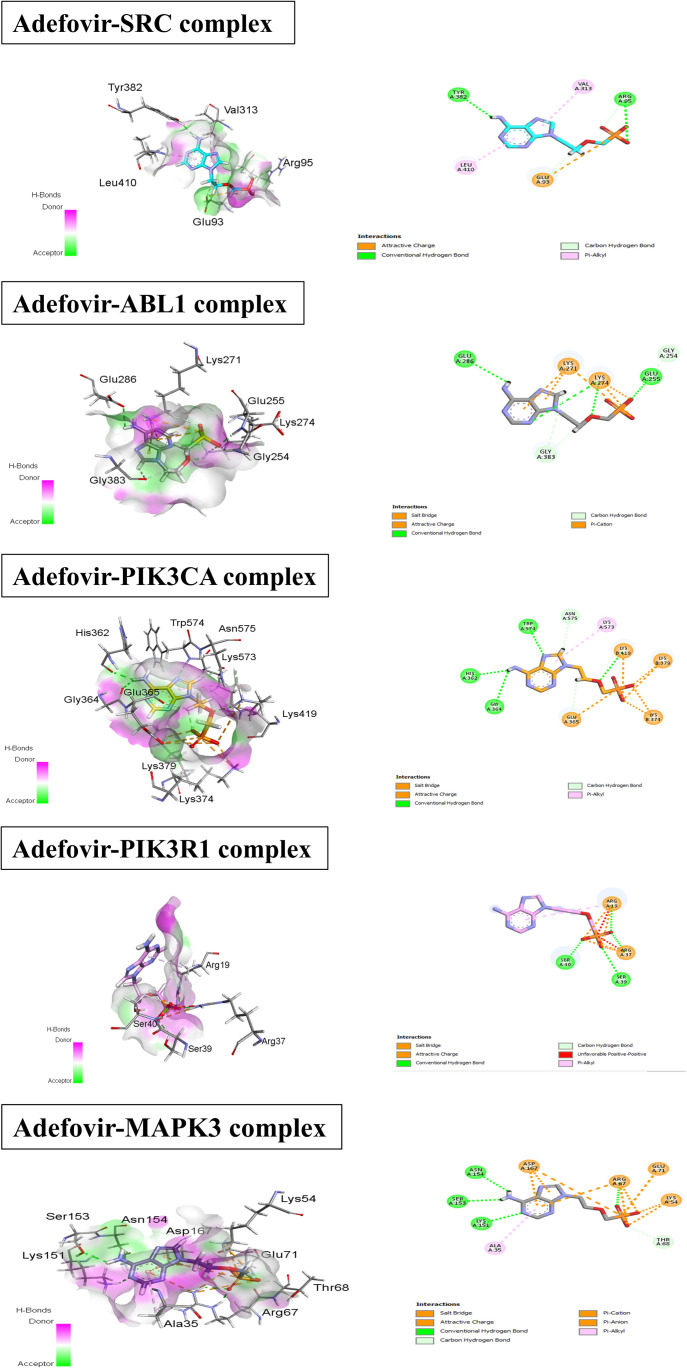
**2D and 3D interactions of adefovir with different cervical cancer proteins.** Where molecular interactions between Adefovir and various protein complexes, including SRC, ABL1, PIK3CA, PIK3R1, and MAPK3 (detail in results). For each complex, specific amino acid residues involved in the interaction are highlighted, such as Tyr382, Val313, Leu410, and Glu93 in the SRC complex, and Glu286, Gly284, and Gly283 in the ABL1 complex. MAPK3: Mitogen-activated protein kinase 3.

### Molecular docking analysis

The molecular docking analysis of adefovir and 5-FU with various target proteins revealed significant differences in binding affinities and interaction characteristics. For the SRC protein, adefovir exhibited a strong binding score of −5.5749 kJ/mol with an RMSD of 1.7828 Å, interacting primarily with residues GLU93, ARG95, TYR382, LEU410, and VAL313. In contrast, 5-FU showed a lower affinity (−4.0853 kJ/mol, RMSD 2.1366 Å) with interactions involving ARG385, ARG409, and ARG419. A similar trend was observed for ABL1, with adefovir scoring −6.0613 kJ/mol (RMSD 1.2009 Å) and interacting with GLU286, GLU255, GLY254, LYS274, and LYS271, compared to 5-FU’s score of −4.1592 kJ/mol (RMSD 0.7213 Å), which interacted with THR272, GLU255, LYS271, and LYS274.For PIK3CA, 5-FU had a score of −4.2951 kJ/mol (RMSD 0.8972 Å) with residues LYS640, ASN677, GLY1007, GLY1009, and SER1008. In contrast, adefovir scored -6.1912 kJ/mol (RMSD 1.1876 Å), interacting with HIS362, GLY364, TRP564, ASN575, LYS573, LYS419, LYS379, LYS374, and GLU365. PIK3R1 showed a score of −4.4766 kJ/mol (RMSD 1.3885 Å) for 5-FU, with interactions involving ARG19, ARG37, LYS41, ALA46, and VAL59. Adefovir, however, scored −5.5194 kJ/mol (RMSD 0.8883 Å) with similar interactions, involving ARG19, ARG37, LYS41, ALA46, and VAL59. Lastly, for MAPK3, adefovir exhibited the highest binding score of −6.2941 kJ/mol (RMSD 2.2148 Å), strongly interacting with LYS151, SER153, ASN154, ALA35, ASP167, ARG67, GLU71, and LYS54. 5-FU, by comparison, had a binding score of −4.3162 kJ/mol (RMSD 0.6837 Å), with interactions involving THR68, LYS54, ARG62, ILE56, GLU71, and ASP167. Overall, adefovir consistently demonstrated stronger binding affinities than 5-FU across all proteins, with the highest affinity observed for MAPK3. This suggests that adefovir could be a more effective therapeutic candidate (Figure 7).

### *In vitro* anti-proliferative activity of adefovir against HeLa cell line

#### Adefovir exhibited cytotoxicity in a dose-dependent manner

**Figure 8.**
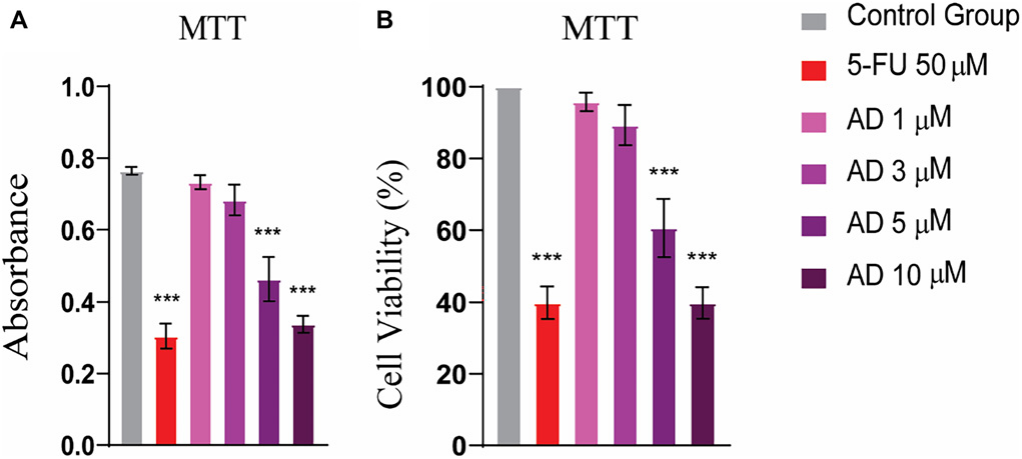
**MTT assay showed strong anticancer potential of adefovir at doses of 5 and 10 µM.** One-way ANOVA followed by Tukey’s multiple comparison test, *n* ═ 3, *** ≤ 0.001. 5-FU: 5-Fluorouracil.

**Figure 9.**
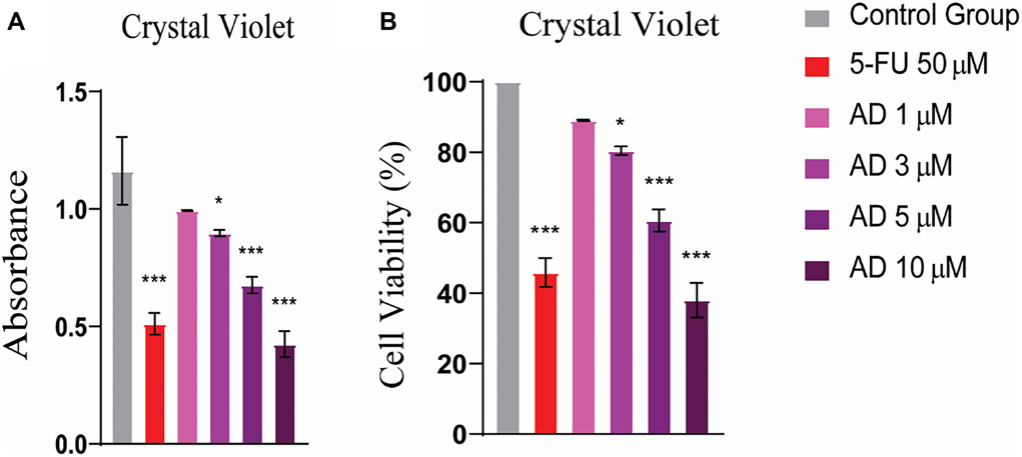
**CV assay revealed significant anti-proliferative activity of adefovir at doses greater than 3 µM.** Tukey’s multiple comparison test after a one-way ANOVA, *n* ═ 3, *** ≤ 0.001. 5-FU: 5-Fluorouracil; CV: Crystal violet.

The MTT assay was performed to evaluate the antiproliferative activity of adefovir against human HeLa cells. HeLa cells were treated with increasing concentrations of adefovir (1, 3, 5, and 10 µM) for 24 h. The results show that as the concentration of adefovir increases from 1 µM to 10 µM, both absorbance and cell viability decrease, indicating that higher doses exert a greater inhibitory effect on cell proliferation, with an IC_50_ value of 7.8 µM (Figure 8). Additionally, a CV assay was conducted to assess cell viability, revealing a more pronounced antiproliferative effect of adefovir on HeLa cells at concentrations greater than 3 µM, compared to 5-FU (Figure 9).

### Adefovir-induced cell death in HeLa cell line

**Figure 10.**
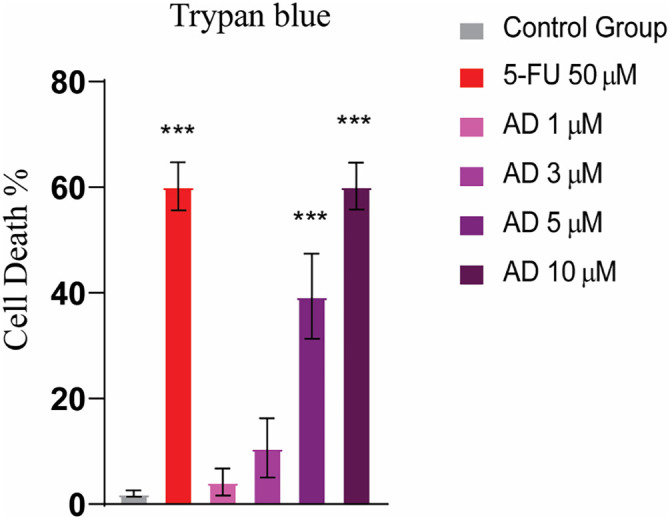
**Trypan blue staining showed an increased cell death after treatment with Adefovir.** Tukey’s multiple comparison test after a one-way ANOVA, *n* ═ 3, *** ≤ 0.001. 5-FU: 5-Fluorouracil.

A trypan blue assay was conducted to assess cell death induced by adefovir. The staining results showed that more than 50% of HeLa cells underwent cell death following adefovir treatment (Figure 10).

### Anti-proliferation through ELISA of VEGF

**Figure 11.**
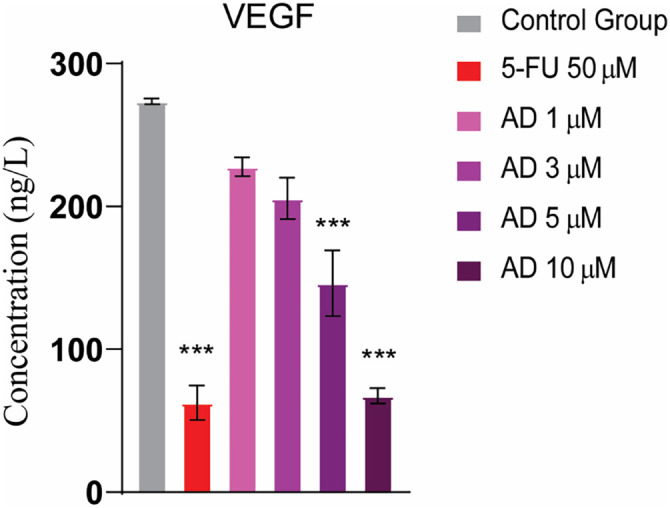
**VEGF activity for different treatment groups illustrates significant differences in treated groups as compared to the control group.** Tukey’s multiple comparison test after a one-way ANOVA, *n* ═ 3, *** ≤ 0.001. 5-FU: 5-Fluorouracil; VEGF: Vascular endothelial growth.

The bar graph illustrates the impact of various treatments on VEGF concentrations. The control group exhibited the highest VEGF levels, around 300 ng/L. Treatment with 5-FU resulted in a modest decrease, lowering VEGF to just over 250 ng/L. Lower doses of the experimental drug adefovir had similar effects, with a 1 µM dose reducing VEGF to roughly 250 ng/L. However, higher doses of adefovir led to a more significant reduction in VEGF, with a dose-dependent response. The 10 µM adefovir treatment produced the most substantial inhibitory effect, reducing VEGF to approximately 50 ng/L (Figure 11).

**Figure 12.**
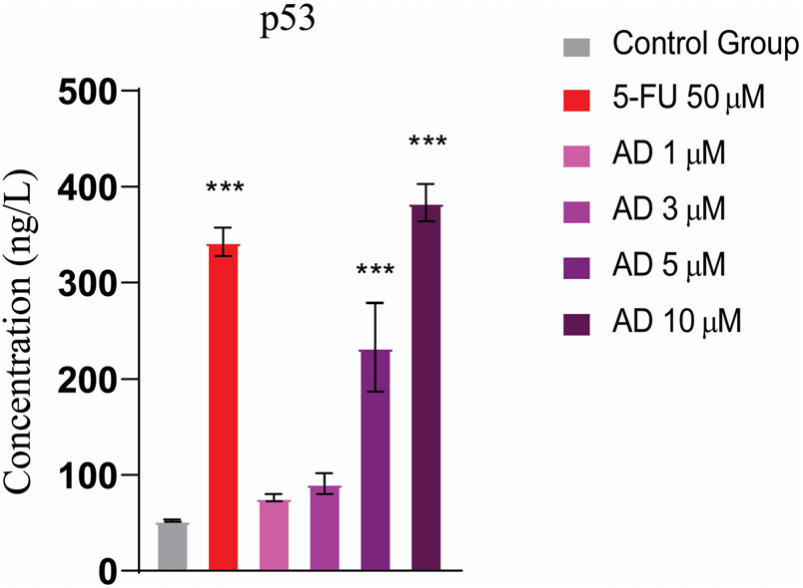
**Expression of p53 in different treatment groups.** The treated groups showed increased levels of p53 compared to the untreated control group. Tukey’s multiple comparison test after a one-way ANOVA, *n* ═ 3, *** ≤ 0.001. 5-FU: 5-Fluorouracil.

**Figure 13.**
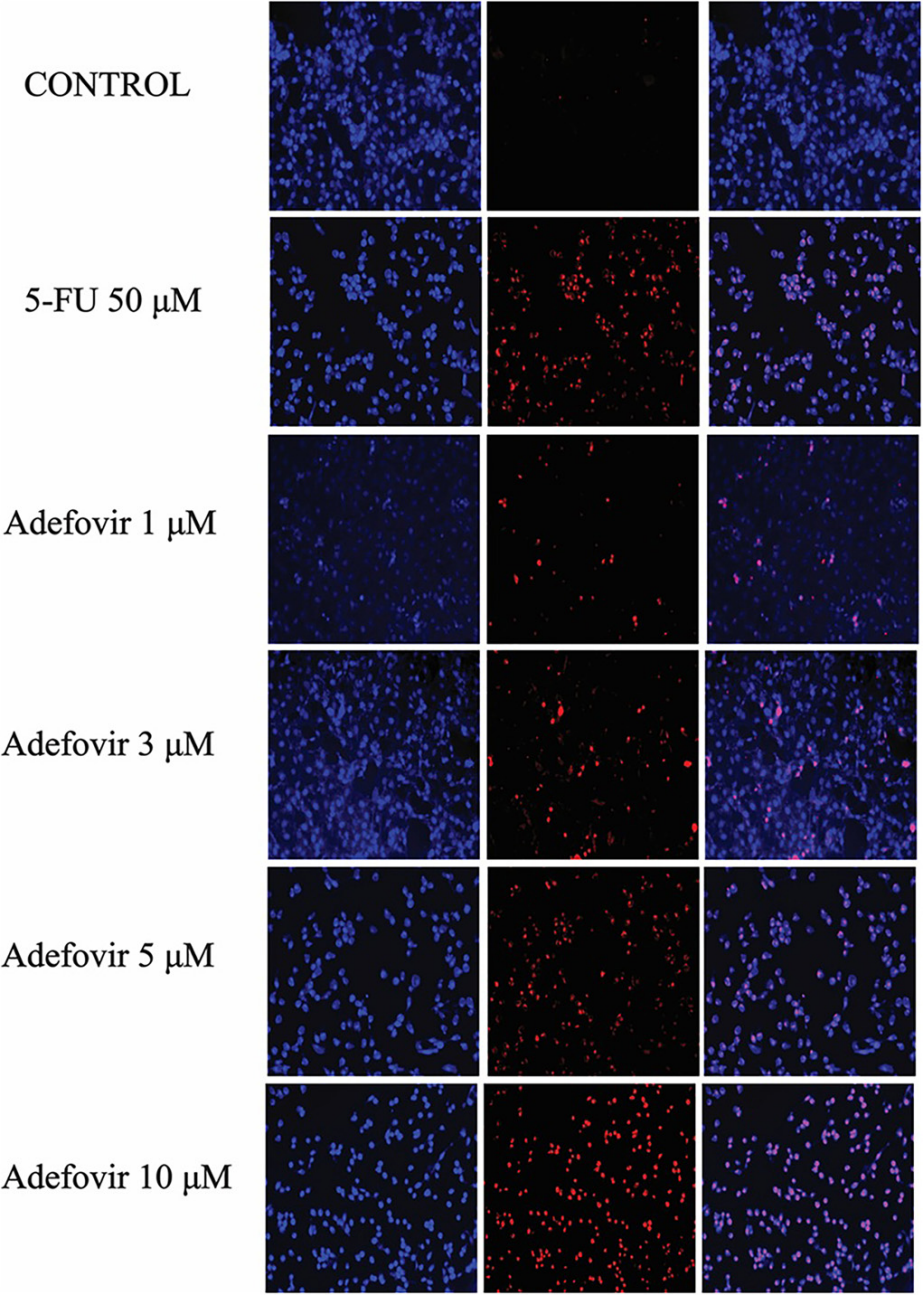
**PI staining depicting increased cell death in adefovir and 5-FU treated groups.** Blue indicates DAPI staining for nuclei, and red indicates PI staining for dead cells. 5-FU: 5-Fluorouracil.

### Apoptosis through ELISA of p53

The bar graph illustrates the concentration of p53 protein across different treatment groups. The control group displayed the lowest levels of p53, while treatment with 50 µM 5-FU led to a significant increase in protein concentrations. Adefovir treatment induced p53 in a dose-dependent manner, with progressively darker shades of purple reflecting higher concentrations from 1–10 µM. Notably, the 5 and 10 µM doses of adefovir significantly increased p53 levels compared to the lower 1 and 3 µM doses. Overall, the data show that both 5-FU and adefovir exposure elevate p53 protein levels relative to the untreated controls (Figure 12).

### PI staining displayed the anti-proliferative potential of Adefovir

DAPI and PI staining revealed significant differences in cell shape and number between control and adefovir-treated cells, indicating stronger anticancer activity of adefovir compared to 5-FU (Figure 13).

## Discussion

Cervical cancer is one of the leading causes of cancer-related deaths among females worldwide [29]. Despite advancements in treatment options, recurrence and the development of resistance remain significant challenges. Therefore, there is an urgent need to explore new therapeutic strategies [30]. In this context, the current study employs a comprehensive network pharmacology approach to investigate the mechanism of action of Adefovir, an anti-hepatitis B virus drug, against cervical cancer. To begin, potential targets of Adefovir and genes associated with cervical cancer were identified using various databases. A total of 182 Adefovir targets and 11,096 cervical cancer-related genes were retrieved. Venn analysis revealed 144 common targets between Adefovir and cervical cancer. These 144 targets were further analyzed through a PPI) network constructed using STRING and visualized in Cytoscape. The resulting PPI network contained 149 nodes and 614 edges, highlighting significant interconnectivity between targets. Key proteins or “hubs” that play crucial roles in cellular processes were identified, including SRC, ABL1, PIK3CA, PIK3R1, and MAPK3 (Figure 6). These proteins are involved in critical signaling pathways that are often deregulated in cancer (Figure 3). Pathway and GO enrichment analysis provided functional insights into the identified targets. Significant enrichment in BPs such as phosphorylation and protein modification suggest that Adefovir may exert its effects by modulating essential signaling mechanisms involved in cancer [31]. Additionally, CC analysis revealed enrichment in focal adhesion and dendrites, suggesting a potential role for Adefovir in cytoskeleton remodeling and cell migration. Taken together, these in silico analyses offer compelling evidence for the anticancer potential of Adefovir and highlight promising therapeutic targets. The next crucial step was to evaluate the direct binding interactions between adefovir and the prioritized targets. The 3D protein structures of the top five hub targets—SRC, ABL1, PIK3CA, PIK3R1, and MAPK3—were retrieved from the PDB (Figure 7). Active binding pockets were identified using CASTp. Molecular docking of adefovir and 5-FU (a standard chemotherapeutic agent) was performed using MOE. Across all targets, adefovir demonstrated consistently stronger binding affinities compared to 5-FU, with the highest binding scores observed for MAPK3 (Table 2). These proteins are involved in critical cancer-related pathways, including SRC in the MAPK signaling pathway [32], ABL1 in JAK/STAT signaling [33], PIK3CA in the PIK3-Akt pathway [34], and MAPK3 in the Ras signaling pathway [35]. These pathways play essential roles in processes, such as proliferation, survival, angiogenesis, and metastasis when dysregulated in cancer. Binding mode analyses revealed that adefovir formed favorable interactions with key residues at the active site. These in silico docking experiments provided preliminary evidence for adefovir’s capability to directly target important cervical cancer proteins [36]. To complement the computational analysis, experimental assessment was carried out using the HeLa cervical cancer cell line model. MTT, CV, and trypan blue assays demonstrated that adefovir potentially inhibited HeLa cell proliferation and induced cell death in a dose-dependent manner, as previously reported by Rafi et al. [37] (Figures 8–12). Remarkably, adefovir showed superior anti-proliferative effects compared to 5-FU at higher concentrations. DAPI/PI staining also revealed morphological changes induced by adefovir treatment. Collectively, these findings validate the anticancer activity of adefovir, as predicted by the network analyses. The observed IC_50_ of 7.8 µM suggests that clinically achievable concentrations are effective. Our study further demonstrated that adefovir treatment significantly increased the expression of p53 in HeLa cells compared to the untreated control group. This observation is supported by a distinct increase in mean p53 expression levels across the treatment groups, as measured by ELISA. P53 is an important tumor suppressor protein, often referred to as the “guardian of the genome” due to its involvement in cell cycle regulation, DNA repair, and apoptosis following cellular stress or DNA damage. In many cancer cells, including HeLa cells, p53 is either mutated or functionally inactivated, allowing for unchecked cell proliferation. Reactivating or upregulating wild-type p53 is, therefore, a promising therapeutic strategy for targeting cancer cells [38, 39].

**Table 2:** Molecular docking analysis of adefovir and 5-FU with different proteins

**Proteins**	**Compounds**	**S score** **(kJ/mol)**	**RMSD** **(Å)**	**Interacting residues**
SRC	Adefovir	−5.5749	1.7828	GLU93,ARG95,TYR382,LEU 410,VAL313
	5FU	−4.0853	2.1366	ARG385,ARG4 09,ARG419
ABL1	Adefovir	−6.0613	1.2009	GLU286,GLU2 55,GLY254,L YS274,LYS27 1
	5FU	−4.1592	0.7213	THR272,GLU2 55,LYS271,L YS274
PIK3CA	Adefovir	−6.1912	1.1876	HIS362,GLY3 64,TRP564,A SN575,LYS57 3,LYS419, LYS379,LYS3 74,GLU365
	5FU	−4.2951	0.8972	LYS640,ASN6 77,GLY1007, GLY1009,SER 1008
PIK3R1	Adefovir	−5.5194	0.8883	SER39,SER40,ARG19,ARG3 7
	5FU	−4.4766	1.3885	ARG19,ARG37,LYS41,ALA4 6,VAL59
MAPK3	Adefovir	−6.2941	2.2148	LYS151,SER1 53,ASN154,A LA35,ASP167,ARG67, GLU71, LYS54
	5FU	−4.3162	0.6837	THR68,LYS54,ARG67,ILE5 6,GLU71,ASP 167

MAPK3: Mitogen-activated protein kinase 3.

Our findings suggest that adefovir, a nucleotide analog primarily used as an antiviral agent, may have potential anticancer properties by modulating p53 expression. Previous research has shown that nucleotide analogs can induce DNA damage, activating p53-dependent pathways. In our study, the increase in p53 expression following adefovir treatment may indicate its role in inducing cellular stress, which in turn activates p53. This activation could lead to cell cycle arrest or apoptosis in HeLa cells, consistent with p53’s known mechanisms of action. Research on plant extracts has explored their potential to counteract the effects of VEGF. These extracts have been shown to effectively reduce VEGF levels, subsequently suppressing angiogenesis. Consistent with this, when cancerous cells were exposed to adefovir treatment, notable reductions in VEGF levels were observed, leading to the inhibition of angiogenesis. Overall, this study employed a network pharmacology approach combined with experimental validation to explore the therapeutic potential of adefovir against cervical cancer. The key findings, mechanistic insights, and promising *in vitro* data suggest that adefovir could be a valuable candidate for further investigation as an alternative or adjunct treatment option.

## Conclusion

In conclusion, this study highlights network pharmacology as a valuable approach for uncovering multi-target mechanisms and expanding the non-oncology applications of approved drugs. The promising preclinical results justify further investigation of adefovir through rigorous *in vivo* evaluations and clinical trials. Successful validation could revolutionize cervical cancer treatment by offering an effective yet safer alternative to conventional chemotherapies. More broadly, the study demonstrates how integrating systems-level analyses with experimental validations can advance drug repurposing for cancer.

## Data Availability

All the data generated in this research work has been included in this manuscript.
